# Epoxy Based Blends for Additive Manufacturing by Liquid Crystal Display (LCD) Printing: The Effect of Blending and Dual Curing on Daylight Curable Resins

**DOI:** 10.3390/polym12071594

**Published:** 2020-07-18

**Authors:** Claudio Tosto, Eugenio Pergolizzi, Ignazio Blanco, Antonella Patti, Paul Holt, Sarah Karmel, Gianluca Cicala

**Affiliations:** 1 Department of Civil Engineering and Architecture (DICAr), University of Catania, Viale Andrea Doria 6, 95125 Catania, Italy; claudio.tosto@unict.it (C.T.); euper@hotmail.it (E.P.); iblanco@unict.it (I.B.); antonella.patti@unict.it (A.P.); 2 Photocentric Ltd., Cambridge House, Oxney Road, Peterborough PE1 5YW, UK; paul.Holt@photocentric.co.uk (P.H.); sarah.karmel@photocentric.co.uk (S.K.)

**Keywords:** additive manufacturing, LCD printing, epoxy, polymer blends, thermomechanical properties

## Abstract

Epoxy-based blends printable in a Liquid Crystal Display (LCD) printer were studied. Diglycidyl ether of bisphenol A (DGEBA) mixed with Diethyltoluene diamine (DETDA) was used due to the easy processing in liquid form at room temperature and slower reactivity until heated over 150 ${}^{{}^{\circ}}$C. The DGEBA/DETDA resin was mixed with a commercial daylight photocurable resin used for LCD screen 3D printing. Calorimetric, dynamic mechanical and rheology testing were carried out on the resulting blends. The daylight resins showed to be thermally curable. Resin’s processability in the LCD printer was evaluated for all the blends by rheology and by 3D printing trials. The best printing conditions were determined by a speed cure test. The use of a thermal post-curing cycle after the standard photocuring in the LCD printer enhanced the glass transition temperature $T_{g}$ of the daylight resin from 45 to 137 ${}^{{}^{\circ}}$C when post-curing temperatures up to 180 ${}^{{}^{\circ}}$C were used. The $T_{g}$ reached a value of 174 ${}^{{}^{\circ}}$C mixing 50 wt% of DGEBA/DETDA resin with the photocurable resin when high temperature cure cycle was used.

## 1. Introduction

Liquid crystal display (LCD) 3D printing is based on the use of LCD displays as imaging system. The key property of LCD printing is that the light shines through the flat LCD panels directly onto the uncured resin. This allows light not to expand and, thus, pixel distortion is less of an issue with LCD printing as it happens with DLP printing. In addition, a full layer can be exposed at the same time and there is no need to scan the photopolymer point-by-point like in SLA. This had the advantage of a faster 3D printing process. In many LCD printers the light comes from an array of UV or LED lamps. Other photocuring 3D printing technologies are based on different principles: digital light processing (DLP) uses a projector to project the image of the cross section of the object to be printed; stereolithography apparatus (SLA) uses a laser beam to scan and cure the layer of the photocuring resins point by point; multi jet printing (MJP) prints the models spraying liquid photosensitive resins from several nozzles and then curing the layer by using an UV lamp [1]. Back in 2015, the company Carbon3D reported the process named CLIP (Continuous Liquid Interface Production) which is based on an oxygen permeable membrane leading to the consecutive printing of each layer and therefore speeding up part’s production. The CLIP technology is also named DLS (Digital Light Synthesis). The key aspects is the use of oxygen permeation to inhibit the radical polymerization allowing fast continuous printing [2]. Carbon3D technology is not based only on the use of oxygen permeation membrane, but also on the availability of different resin chemistries ranging from acrylates to epoxies, cyanates and polyurethanes. Some of the best performing resins offered by Carbon3D are based on dual curing approach in which photocuring is followed by thermal curing [3]. Obst et al. [4] discussed the influence of different exposure times on the dual curing reaction of a polyurethane-based resin used in the DLS technology. The resin used in their study was a blend of acrylate and polyurethane. The mechanical characteristics such as tensile strength and elongation at break were shown to be strongly dependent on the degree of UV crosslinking. Redmann et al. [5] focused on an epoxy-based system processable in the Carbon3D printer. The authors addressed the problem of thermal curing optimization for this system. Reducing the thermal curing time while avoiding a negative influence on the final mechanical properties was the main goal. The original curing time with a duration of 750 min was shortened to 200 min with, at the same time, an improvement of glass temperature transition from 146 to 154 ${}^{{}^{\circ}}$C. Dual curing for acrylate-based systems has been widely studied for several applications: shape memory polymers, optical materials, photolithography, protective coatings, structured surface topologies, and holographic materials [6]. A dual-curing process is defined as a combination of two curing reactions taking place simultaneously or sequentially. Epoxies mixed with a photocurable acrylic resin cured combining photocuring with thermal curing allowed the 3D printing of carbon fiber reinforced composites [7]. Lantean et al. [8] proposed similar approach based on epoxy/acrylate blends for DLP printing. In this study, epoxy was used but no specific thermally activated curing agent was added. The maximum $T_{g}$ achieved was limited to 108.6 ${}^{{}^{\circ}}$C for those blends. Daylight resins cure at 460 nm while most of the other photocurable resins cure in the UV region using wavelengths between 325–420 nm. According to Photocentric, “the use of light at longer wave length proved to allow for a deeper penetration depth into the photopolymer material and therefore a more uniform and accurate 3D printing process” [9,10]. The combination of visible light with the use of LCD screens for 3D printing, allows for a faster, more efficient and economical manufacturing process. This is a relevant point for application. In the literature there are increasing examples of the use of additive manufacturing in several fields ranging from microfluidics [11,12,13], tooling for composites [14,15] and injection molding [16]. Mixing epoxy blends with photocurable resins can open the field to novel formulation too if complex toughened epoxy blends area used [17,18] because this can be a suitable method to obtain the impact resistance of the printed specimens. All these applications might benefit of an accurate control of cavity control [19,20]. The aim of this work was to improve the thermal properties of daylight resins by blending with epoxy thermally curable resins without altering the working principle of the printer. To achieve this goal first we selected a commercial daylight curing resin and we studied its thermal curing properties after LCD printing. The effect of epoxy blending and the use of dual curing as a mean to improve resin’s thermal properties was analyzed.

## 2. Experimental

### 2.1. Materials and Method

#### 2.1.1. Materials

Two resin systems were mixed: the commercial daylight resin (Cream Hard) obtained by Photocentric Ltd. (Peterborough, UK) and the thermally curable epoxy resin formulated by mixing diglycidyl ether of bisphenol A (DGEBA) and diethyltoluene diamine (DETDA. In the following, we refer to the Cream Hard resin as cream or C while the mixture DGEBA/DETDA is refereed as E. The diglycidyl ether of bisphenol A selected was the Araldite GY 240 kindly donated by Huntsman (Basel, Switzerland). The diethyltoluene diamine (DETDA) was the LonzaCure DETDA kindly donated by Lonza (Basel, Switzerland). DGEBA and DETDA were mixed at room temperature with a 1:1 stoichiometric ratio.

#### 2.1.2. Epoxy-Based Blend Preparation

LonzaCure DETDA is a liquid aromatic amine that can be mixed at room temperature with liquid epoxy. The liquid epoxy formulation obtained is miscible with daylight resins and it is not reactive under daylight. The uncured epoxy formulation was obtained mixing the two monomers (i.e., DGEBA and DETDA) in a centrifugal planetary mixer (ARV-310 by Thinky, Laguna Hills, CA, USA). The conditions used for mixing were: speed 2000 rpm, vacuum pressure of 0.3 kPa and mixing time of 5 min. The epoxy-based blends were prepared by mixing at room temperature the uncured epoxy formulation with the cream resin with different weight ratios (Table 1). The blends were mixed in the centrifugal planetary mixer with the same mixing procedure used for the uncured epoxy formulation. The prepared blends were stored in opaque glass containers avoiding daylight exposure until used. No sign of demixing or incompatibility for the samples stored at room temperature for 3 months was observed. The centrifugal mixer allowed to mix the two resins in 5 min only and, at the same time, to apply deareation avoiding air voids in the cured systems.

#### 2.1.3. Resin LCD Printing and Thermal Curing

The LCD printing was carried out on a LC-HR2 LCD printer by Photocentric (Peterborough, UK). The LC-HR2 is a LCD printing machine that uses an iPad screen as display for resin’s photocuring. The initial conditions used for printing the cream resin were recommended by Photocentric. Following the printing trials on the epoxy- based blends the printing conditions were tailored to obtain good quality printing (Table 2). The methods used to define the optimized printing parameters for the epoxy blends will be explained in the results and discussion section. The printed samples were first shaped by LCD printing and then thermally post-cured in a standard ventilated oven with two types of cure cycles: isothermal cure for hold time of 3 h; isothermal cure at 140 ${}^{{}^{\circ}}$C for 2 h followed by a ramp at 2 ${}^{{}^{\circ}}$C/min to 180 ${}^{{}^{\circ}}$C and hold at this temperature for 2 h. For the isothermal curing of the cream resin three temperatures were tested: 100, 140 and 150 ${}^{{}^{\circ}}$C.

### 2.2. Characterization

#### 2.2.1. Dynamic Mechanical Analysis (DMA)

Cured specimens with dimensions (10 × 8 × 2) ${\mathrm{mm}}^{3}$ were tested using a dynamic mechanical analysis (DMA) machine (Tritech by Triton Ltd., Wrexham, UK) to measure the storage modulus (*E’*) and the *Tanδ*. The experiments were carried out in single cantilever mode with a 20 μm amplitude and 1 Hz frequency. Two types of experiments were carried out: isothermal testing by heating the DMA chamber at 10 ${}^{{}^{\circ}}$C/min followed by an isothermal hold for 120 min at different temperatures (i.e., 100, 140 and 150 ${}^{{}^{\circ}}$C); ramp test at 2 ${}^{{}^{\circ}}$C/min from 25 to 180 ${}^{{}^{\circ}}$C. The isothermal test was carried out on the cream sample after LCD printing to evaluate the effect of the thermal crosslinking induced by postcuring. The ramp rate test was used to characterize the viscoelastic properties of the samples after completing the thermal curing cycle. The latter test was used to measure the glass transition temperature ($T_{g}$ ) of the resin that was determined as the *Tanδ* peak.

#### 2.2.2. Differential Scanning Calorimetry (DSC)

Calorimetric measurements were performed using a Shimadzu DSC-60 (Shimadzu, Kyoto, Japan). Resin samples in liquid (uncured resin) and solid (photo- and thermal- cured resin) form were placed in a 40-μL sealed aluminum crucibles. Samples of the average weight of 5–7 mg have been prepared. The samples were heated from room temperature up to 300 ${}^{{}^{\circ}}$C at a rate of 5 ${}^{{}^{\circ}}$C/min (first scan). After the first scan, they were cooled to room temperature and reheated to 300 ${}^{{}^{\circ}}$C, at a heating rate of 5 ${}^{{}^{\circ}}$C/min (s scan). The glass transition temperature ($T_{g}$) was measured as the midpoint of the heat capacity increment Δ*c*p associated with the glass–rubber transition. The specific heat increment Δ*c*p was calculated from the vertical distance between the two extrapolated baselines at the glass transition temperature. The total exothermic heat released was calculated from the area of the DSC exothermic peak.

#### 2.2.3. Rheology Testing

Parallel plate rheometry was carried out on uncured resin blends using an ARES rheometer (TA Instruments, Sesto San Giovanni (Mi), Italy). Disc-disc configuration was used with plates of 25 mm diameter and 1.2 mm gap between plates. Isothermal test at room temperature with strain of 5% and oscillatory frequency of 10 Hz were carried out on the blends.

## 3. Results and Discussion

### 3.1. DSC Testing Results

**Figure 1:**
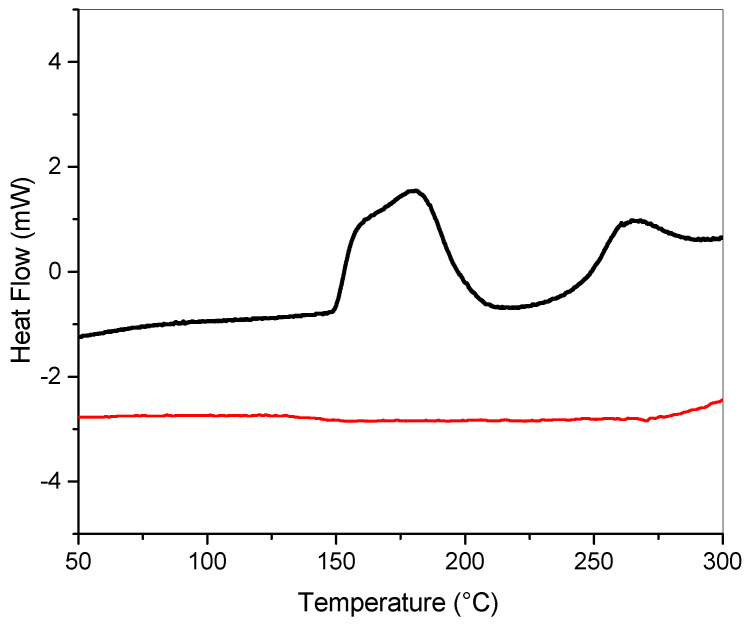
DSC analysis on the hard cream resin: first scan (black); second scan (red).

The uncured cream resin was tested before LCD printing by a dynamic temperature scan using differential calorimetry. The result is reported in Figure 1. The graph shows the presence of an exothermic peak centered at 180 ${}^{{}^{\circ}}$C starting at 150 ${}^{{}^{\circ}}$C (black curve). The total exothermic heat released was equal to 168.35 J/g. The exothermic peak reveals the presence of some thermally reactive groups in the daylight formulation. The cream resin is a (meth)acrylate-based resin that can be cured by photopolymerization and by heat [21]. The cream resin was analyzed back after the first scan and no exothermic peak was observed (Figure 1, red curve). This finding confirms that all reactive moieties were reacted in the first scan. The thermogram clearly shows a glass transition temperature at 135.6 ${}^{{}^{\circ}}$C in the second scan (red curve) that is higher than the $T_{g}$ reported for the cream resin after LCD curing only (i.e., ≈60 ${}^{{}^{\circ}}$C). Such an improvement can be explained as the result of the increased crosslink density caused by the thermal curing occurring in the first scan.

**Figure 2:**
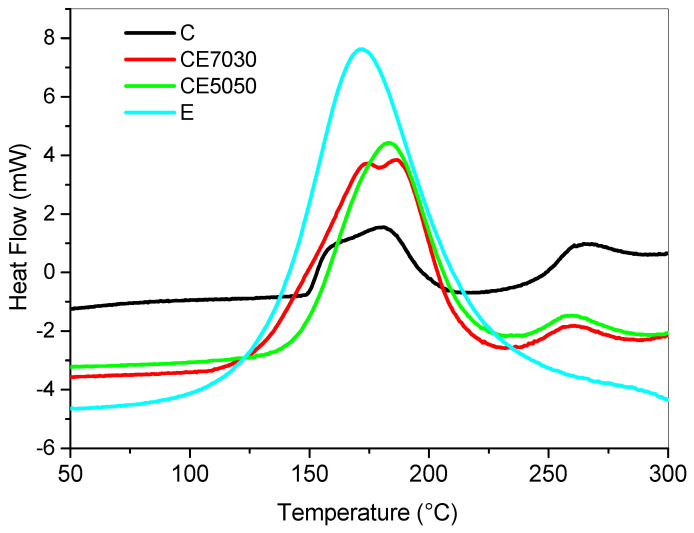
DSC analysis on the epoxy-based blends in their uncured state.

The unreacted epoxy-based blends were also characterized in their uncured state by DSC. The results are summarized in Figure 2. The addition of the thermally curable resin resulted in the increase of the released heat during the heating ramp (Table 3) as it can be expected because of the presence of a higher content of thermally curable monomers.

### 3.2. Rheology Testing

**Figure 3:**
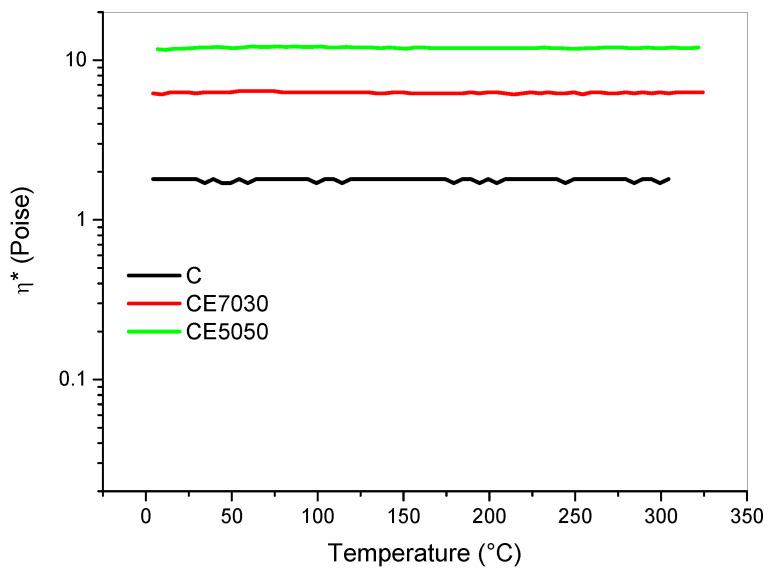
Complex viscosity of the uncured epoxy-based blends: C (blue); CE7030 (red); CE5050 (green).

Resin viscosity is a parameter of paramount importance for LCD printing. The resin must flow during platform movement up and down in order to create a homogenous uncured resin layer between the bottom of the vat and the printing part. Photocentric suggests a maximum viscosity (η*) of 100 Poise to enable resin printing on the LC-HR2 printer. The commercial cream resin used in this study presented a room temperature viscosity of about 1.8 Poise. Adding the epoxy formulation resulted in a viscosity increase with increasing epoxy content. The blend with the highest content of epoxy (i.e., CE5050) showed a room temperature viscosity of 12 Poise (Figure 3). The blend’s viscosity largely depends on the epoxy prepolymer molecular weight and, as such, could be tailored by the addition of reactive diluents. We limited blend’s viscosity at values within printer specifications and at the same time we increased the $T_{g}$ values. Notably, the $T_{g}$ values achieved are higher then the values obtained by most of the current commercially available systems by Photocentric and they are also better that other commercial systems (Table S1). The resins which displayed similar performance where those sold by Carbon3D and based on the dual curing processing. If we compare the $T_{g}$ results and the full processing times we obtained with the results presented on Carbon3D technology in the open literature [2,4,5] we can notice that proposed approach is fast and lead to comparable $T_{g}$ performance. The viscosity of the blends studied in this paper are higher than those of the systems commercially available. However, being the values well below the suggested limit of 100 Poise the blends were all regarded as being processable and this was confirmed by our printing trials described in the following section.

### 3.3. Printing Test

**Figure 4:**
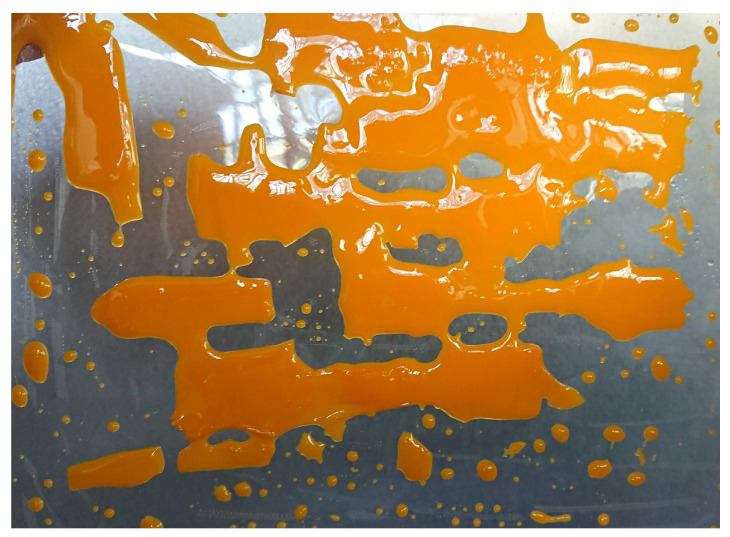
Printing trials for the CE7030 blend with the same printing conditions used for the cream resin (see Table 2).

Once tested blend’s viscosity, printing trials to obtain specimens for thermomechanical testing were carried out. The first trials on the CE7030 blend resulted in unsuccessful printing (Figure 4). The printing failed completely as no shape was formed and only some cured layers deposited on the vat films were found. The same printing parameters used for the cream resin were applied in such trials (see Table 2). The short exposure times seemed not long enough to allow full curing of resin’s layers properly. This result is the effect of two parameters: the presence in the blends of the epoxy resin, which is designed for thermal curing only, that reduce the amount of photocurable moieties; the stoichiometric unbalance of the photointiator due to the epoxy resin addition in the blend formulation.

To optimize the exposure times of the formulated blends the photocuring of one single layer was studied in greater detail using the following procedure. The printer was set to print a single layer prism with a square base of 10 mm. One single layer for the LC-HR2 printer would results in a layer thickness of 100 μm when the cream resin is used. The uncured blend was dropped on a plastic foil and suddenly exposed to the LCD light with different exposure times setting the display to project one layer only for the prism. The LC-HR2 printer works with fixed light intensity therefore this parameter was not investigated. The uncured resin was gently rinsed away with a cloth wetted with isopropyl alcohol (IPA) after light irradiation. The resulting thickness of the printed prism was measured by a caliper. The side of the resulting square prism was measured by a caliper and the deviation from the fixed side (i.e., 10 mm) reported as width overcure (Figure S2). The obtained results are summarized in see Table 4. The results shown in the table demonstrate that adding the epoxy resin to the photocurable resin there is a slightly reduced photo-reactivity for the blend. Therefore, to account for such effect the exposure times increased from 25 s of the cream resin to 30 s for the CE7030 and CE5050 (Figure Table 2). However, the printing results is also affected by the resin flow during the up and down movement of the platform. To account for the increased viscosity of the blends (Figure 3) the platform’s controlled movement were modified as reported in Table 2 for the blends. In particular, the platform was lifted higher to 10 mm distance instead of the 3 mm used for the cream and, to ensure the correct timing for resin flow, the Z lift and retract were slower (i.e., 10 mm/min) compared to the cream resin’s printing.

**Figure 5:**
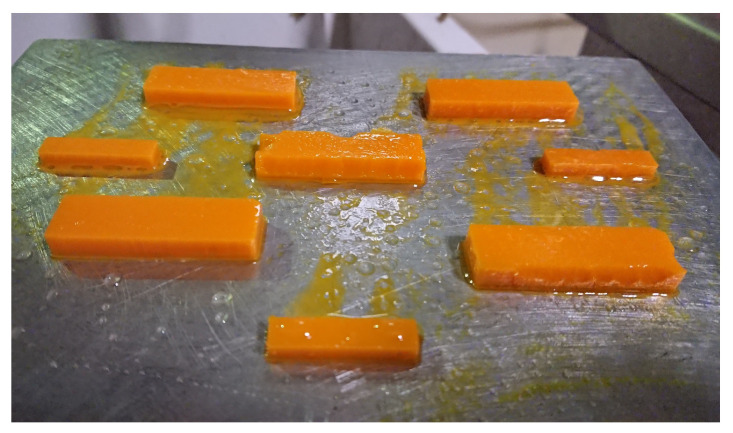
Printing trials for the CE7030 blend with the optimized printing conditions after photocuring optimization (see Table 2).

Following these findings new settings were used Table 2 that yielded good quality specimens as all the shapes drawn were printed (Figure 5) and all the samples had the dimension planned.

### 3.4. DMA Testing

**Figure 6:**
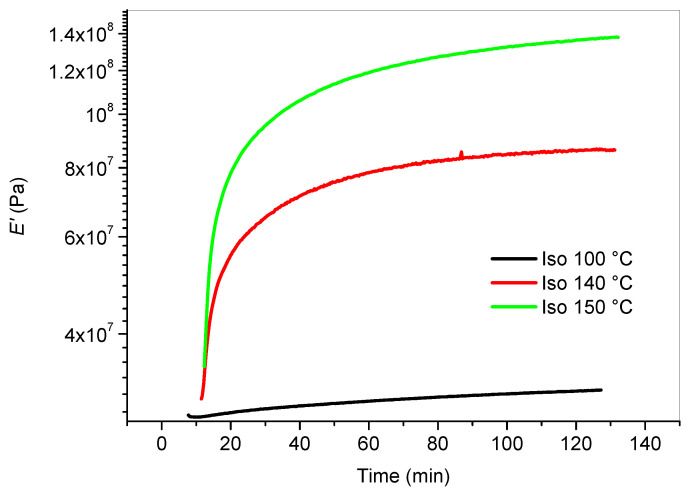
Isothermal DMA curing test on LCD printed Hard Cream specimens: Storage modulus versus time.

The results of the DSC tests showed the presence of an exothermic peak for the uncured cream resin outlining the presence of reactive moieties leading to the thermal curing of this system (see Figure 1). DMA is a suitable technique for the study of the thermal curing of solid systems after vitrification [22]. LCD photo-cured samples were analyzed by running isothermal DMA testing at three different temperatures (i.e., 100, 140 and 150 ${}^{{}^{\circ}}$C). This test allowed to measure viscoelastic properties changes during the isothermal curing. Storage modulus (*E’*) versus time was monitored in detail. The cream resin tested at 100 ${}^{{}^{\circ}}$C for 3 h showed only a slight variation of the storage modulus while, when the isothermal test was carried out at 140 or 150 ${}^{{}^{\circ}}$C, the storage modulus increased of one order of magnitude within the testing time (Figure 6). The increase of the storage modulus is the result of the crosslinking reactions which starts, as demonstrated by the DSC tests (Figure 1), at 150 ${}^{{}^{\circ}}$C.

**Figure 7:**
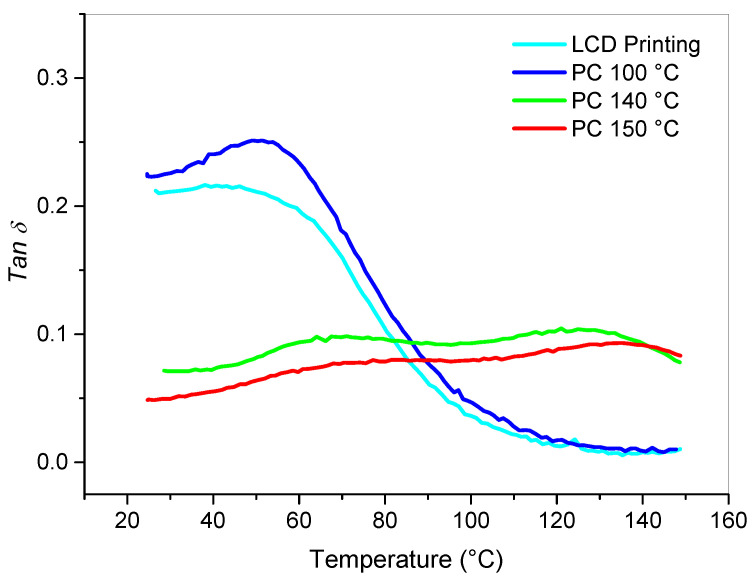
Ramp rate DMA test on isothermally cured Hard Cream resin.

After being tested isothermally, the same samples were further analyzed by a ramp rate DMA test (Figure 7). The system cured at 140 ${}^{{}^{\circ}}$C displayed two *Tanδ* peaks at 65 and 126 ${}^{{}^{\circ}}$C. The resin cured at 150 ${}^{{}^{\circ}}$C displayed one peak at 134 ${}^{{}^{\circ}}$C and a shoulder at about 67 ${}^{{}^{\circ}}$C. The specimen cured at 100 ${}^{{}^{\circ}}$C showed a slight shift of the *Tanδ* peak from 45 ${}^{{}^{\circ}}$C, measured for the LCD printed sample, to 50 ${}^{{}^{\circ}}$C.

**Figure 8:**
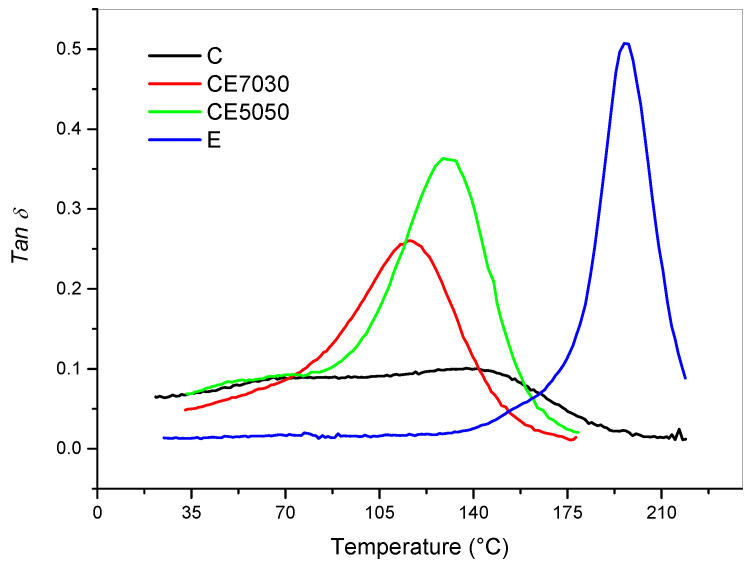
DMA analysis for the LCD printed sample after the post-curing cycle (isothermal cure at 140 ${}^{{}^{\circ}}$C for 2 h, ramp at 2${}^{{}^{\circ}}$C/min to 180 ${}^{{}^{\circ}}$C and hold at 180 ${}^{{}^{\circ}}$C for 2 h).

The shift of $T_{g}$ peaks, which corresponds to the glass transition temperature ($T_{g}$), is the result of the increased crosslink density due to the crosslinking reactions occurring because of the thermal curing of the cream resin. The epoxy DGEBA/DETDA system showed an exothermic peak centered at 172 ${}^{{}^{\circ}}$C (Figure 2). Therefore, to ensure the full conversion of the epoxy moieties after LCD printing the following post-cure cycle for epoxy modified blends was used: isothermal cure at 140 ${}^{{}^{\circ}}$C for 2 h, ramp at 2 ${}^{{}^{\circ}}$C/min up to 180 ${}^{{}^{\circ}}$C and hold at 180 ${}^{{}^{\circ}}$C for 2 h. The post- cured samples obtained were analyzed by a ramp test (Figure 8).

**Figure 9:**
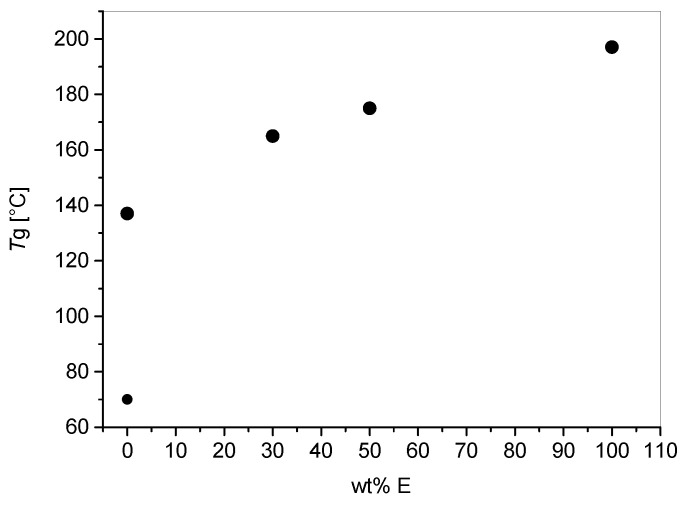
Glass transition temperatures ($T_{g}$) obtained as *Tanδ* peaks measured by DMA plotted versus the epoxy content.

As with the viscosity, if we can consider commercial analogous systems (Table S1) it can be said that with dual curing, resin with higher $T_{g}$ were obtained. The cream unmodified resin (C) showed two glass transition temperatures ($T_{g}$) at 70 and 137 ${}^{{}^{\circ}}$C while the pure epoxy formulation (E) a $T_{g}$ of 197 ${}^{{}^{\circ}}$C. Epoxy blending resulted in one single broad *Tanδ* peak reaching, for the 50:50 blend, with a $T_{g}$ value of 174 ${}^{{}^{\circ}}$C. The blends obtained mixing the two formulations showed an increasing $T_{g}$ with higher epoxy content (Figure 9). The $T_{g}$ vs epoxy content graph shows a quasi linear correlation considering only the high $T_{g}$ for the cream resin. This trend is typically observed for blends forming the so-called interpenetrated network [23].

## 4. Conclusions

Daylight curable (meth)acrylate-epoxy blends were studied. Calorimetric analysis demonstrated the presence of thermally reactive moieties for the daylight resin used. Dynamic mechanical analysis showed that LCD printed parts do not develop fully crosslinked network coherently with the information gained from DSC analysis. Treating with thermal postcure the LCD printed daylight resins increased the glass transition temperature from 45 to 137 ${}^{{}^{\circ}}$C. This $T_{g}$ increase was obtained with a post treatment that required about 4 h and 20 min of additional thermal curing time. This additional treatment time is not impacting dramatically on overall production times because LC-HR2 printer can print 100 μm every 25 s thus leading to a printing time of 7 h for a complex part with a volume of 78 ${\mathrm{cm}}^{3}$ (Figure S1). Regarding this point, we should notice that increasing the printing area on LCD printer do not change the printing time. Therefore, if many similar objects are printed in bigger printers (like for example the Magna model) the printing time remains approximately the same while the curing time can be the same on all the parts. This means that the time to make parts can be overall reduced because of post-curing is carried out at once on all parts. These concepts let Photocentric to achieve high volume throughputs with their big area machines. Therefore, thermal post-curing of daylight resin is an efficient approach for enhancing the final properties of LCD printed parts. The benefits of thermal post-curing were further enhanced when DGEBA/DETDA resin was added to the formulation. The $T_{g}$ increased up to 165 and 174 ${}^{{}^{\circ}}$C for blends with 30 and 50 wt% of DEGBA/DETDA, respectively. However, these remarkable $T_{g}$ increases showed some drawbacks due to the increasing photocuring times to achieve good quality LCD printing. The exposure time needed to be adjusted from 25 s for hard cream resin to 30 s for the epoxy-based blends and the platform to be moved higher and slower to allow resin flow. The changes in these printing parameters lead to longer printing time. For example, the part (Figure S1) with a volume of 78 ${\mathrm{cm}}^{3}$ would be of 16 h of printing time. These results offer some important insights for the further development of similar blends. To counteract the reduced photocuring reactivity of the modified blends some adjustments of the type and content of the photoinitiator could a viable solution while, blend’s viscosity could be tailored by using reactive diluents. Such developments can be interesting for future applications in the field of tooling for polymers and composites or for specific application in severe environment. In the field of tooling for polymers and composites the need for high temperature resistance is due to the high processing temperatures. For example, many advanced prepregs needed in automotive are cured with processing cycles at 120 ${}^{{}^{\circ}}$C thus requiring materials with high temperature stability. Other fields which might benefit for the use of high Tg resins and for the high resolution offered by LCD printing are the valves working with hot fluids. Similar applications for dual curable epoxies have been presented by Carbon3D but, according to the result of this paper, LCD printing can be used too if the epoxy blending approach is pursued.

## Figures and Tables

**Table 1:** Resin formulations studied.

ID Sample	Cream [wt%]	Epoxy [wt%]
C	100	0
CE7030	70	30
CE5050	50	50
E	0	100

**Table 2:** LCD printing conditions used for specimens manufacturing.

ID Sample	Exposure Time [s]	Z Lift Distance [mm]	Z Lift Speed [mm/min]	Z Retract Speed [mm/min]	Top Time [s]
C	25	3	15	50	5
CE7030	30	10	10	10	10
CE5050	30	10	10	10	10

**Table 3:** Data obtained from the DSC analysis of the uncured blends.

ID Sample	Uncured	Photocured	Postcured in Oven	Isothermal [${}^{{}^{\circ}}$C]	Peak${}_{1}$ [${}^{{}^{\circ}}$C]	Onset${}_{1}$ [${}^{{}^{\circ}}$C]	Heat${}_{1}$ [J/g]	Peak${}_{2}$ [${}^{{}^{\circ}}$C]	Onset${}_{2}$ [${}^{{}^{\circ}}$C]	Heat${}_{2}$ [J/g]
E	•				171.79	129.59	264.52	-	-	-
CE5050	•				183.23	148.69	174.53	259.3	253	9.88
CE7030	•				186.56	143.7	227.98	261.06	243.93	9.31
C	•				181	150.24	168.35	264.83	245.33	41.4
C		•			185.56	183.25	115.71	-	-	-
C		•	•	100	188.22	184.52	55.16	-	-	-
C		•	•	140	189.54	185.71	12.05	-	-	-
C		•	•	150	-	-	-	-	-	-

**Table 4:** Average mean values and standard deviations (Std Dev) of the printing trials on the square prism for the LCD printing optimization with the epoxy-based blends.

Exposure Time [s]	Thickness [μm]						Width Overcure [%]					
	C		CE7030		CE5050		C		CE7030		CE5050	
	Mean	Std Dev	Mean	Std Dev	Mean	Std Dev	Mean	Std Dev	Mean	Std Dev	Mean	Std Dev
5	-	-	-	-	-	-	-	-	-	-	-	-
10	32	1.0	-	-	-	-	−9.4%	0.14%	-	-	-	-
15	46	0.7	41	1.0	-	-	−8.1%	0.15%	−9.0%	0.17%	-	-
20	82	1.0	73	1.2	-	-	−4.7%	0.19%	−5.3%	0.20%	-	-
25	105	0.9	95	1.3	88	0.8	0.0%	0.14%	−2.0%	0.15%	−4.0%	0.13%
30	117	1.0	108	1.2	102	1.0	1.1%	0.20%	1.5%	0.12%	1.0%	0.19%
35	137	0.7	125	1.2	118	0.9	1.1%	0.16%	1.6%	0.16%	1.5%	0.12%

## Supplementary Materials

The following are available online at https://www.mdpi.com/2073-4360/12/7/1594/s1.

## Abbreviations

The following abbreviations are used in this manuscript:

η*	Complex viscosity
DETDA	Diethyltoluene diamine
DGEBA	Diglycidyl ether of bisphenol A
DMA	Dynamic Mechanical Analysis
DSC	Differential Scanning Calorimetry
*E’*	Storage modulus
Δ*c*p	Heat capacity increment
LCD	Liquid Crystal Display
$T_{g}$	Glass-transition temperature

## References

[1] Quan H., Zhang T., Xu H., Luo S., Nie J., Zhu X. (2020). Photo-curing 3D printing technique and its challenges. Bioact. Mater..

[2] Tumbleston J.R., Shirvanyants D., Ermoshkin N., Janusziewicz R., Johnson A.R., Kelly D., Chen K., Pinschmidt R., Rolland J.P., Ermoshkin A. (2015). Continuous liquid interface production of 3D objects. Science.

[3] Xinyu G.U., Poelma J., Rolland J.P. (2017). Methods Of Making Three Dimensional Objects From Dual Cure Resins With Supported Second Cure.

[4] Obst P., Riedelbauch J., Oehlmann P., Rietzel D., Launhardt M., Schmölzer S., Osswald T.A., Witt G. (2020). Investigation of the influence of exposure time on the dual-curing reaction of RPU 70 during the DLS process and the resulting mechanical part properties. Addit. Manuf..

[5] Redmann A., Oehlmann P., Scheffler T., Kagermeier L., Osswald T.A. (2020). Thermal curing kinetics optimization of epoxy resin in Digital Light Synthesis. Addit. Manuf..

[6] Konuray O., Fernández-Francos X., Ramis X., Serra À. (2018). State of the Art in Dual-Curing Acrylate Systems. Polymers.

[7] Griffini G., Invernizzi M., Levi M., Natale G., Postiglione G., Turri S. (2016). 3D-printable CFR polymer composites with dual-cure sequential IPNs. Polymer.

[8] Lantean S., Roppolo I., Sangermano M., Pirri C.F., Chiappone A. (2018). Development of New Hybrid Acrylic/Epoxy DLP-3D Printable Materials. Inventions.

[9] Holt P., Zaregadeh H., Karmel S. (2017). Methods for Making a Metal, Sand Or Ceramic Object by Additive Manufacture and Formulations for Use in Said Methods.

[10] Zhu J., Zhang Q., Yang T., Liu Y., Liu R. (2020). 3D printing of multi-scalable structures via high penetration near-infrared photopolymerization. Nat. Commun..

[11] Cairone F., Davi S., Stella G., Guarino F., Recca G., Cicala G., Bucolo M. (2020). 3D-Printed micro-optofluidic device for chemical fluids and cells detection. Biomed. Microdev..

[12] Richard C., Neild A., Cadarso V.J. (2020). The emerging role of microfluidics in multi-material 3D bioprinting. Lab Chip..

[13] Waheed S., Cabot J.M., Macdonald N.P., Lewis T., Guijt R.M., Paull B., Breadmore M.C. (2016). 3D printed microfluidic devices: Enablers and barriers. Lab Chip.

[14] Tosto C., Latteri A., Pergolizzi E., Giordano D., Abramo G., Catenaro R., Pignotti N., Cicala G. (2020). Additive Manufacturing of Plastics: An Efficient Approach for Composite Tooling. Macromol. Symp..

[15] Li H., Taylor G., Bheemreddy V., Iyibilgin O., Leu M., Chandrashekhara K. (2015). Modeling and characterization of fused deposition modeling tooling for vacuum assisted resin transfer molding process. Addit. Manuf..

[16] Kampker A., Ayvaz P., Lukas G. (2020). Direct Polymer Additive Tooling—Economic Analysis of Additive Manufacturing Technologies for Fabrication of Polymer Tools for Injection Molding. Key Eng. Mater..

[17] Cicala G., Recca G., Carciotto S., Restuccia C. (2009). Development of epoxy/hyperbranched blends for resin transfer molding and vacuum assisted resin transfer molding applications: Effect of a reactive diluent. Polym. Eng. Sci..

[18] Cicala G., Mamo A., Recca G., Restuccia C.L. (2007). Study on epoxy/thermoplastic blends based on the addition of a novel aromatic block copolymer. Polym. Eng. Sci..

[19] Sapuppo F., Bucolo M., Intaglietta M., Fortuna L., Arena P. (2006). A cellular nonlinear network: Real-time technology for the analysis of microfluidic phenomena in blood vessels. Nanotechnology.

[20] Cairone F., Gagliano S., Bucolo M. (2016). Experimental study on the slug flow in a serpentine microchannel. Exp. Ther. Fluid Sci..

[21] Maffezzoli A., Terzi R., Nicolais L. (1995). Cure behaviour of visible light activated dental composites. J. Mater. Sci. Mater. Med..

[22] Menard H.P. (2008). Dynamic Mechanical Analysis: A Practical Introduction.

[23] Campbell J.A., Inglis H., Ng WeiLong E., McKinley C., Lewis D.A. (2019). Morphology Control in a Dual-Cure System for Potential Applications in Additive Manufacturing. Polymers.

