# Supplementary material for: Epoxy Based Blends for Additive Manufacturing by Liquid Crystal Display (LCD) Printing: The Effect of Blending and Dual Curing on Daylight Curable Resins
Source: Polymers (Basel). 2020 Jul 18;12(7):1594. doi: 10.3390/polym12071594 (PMC7408060; doi:10.3390/polym12071594)
Supplement: Supplementary file 1 [file polymers-12-01594-s001.pdf]

Supplementary Materials: Epoxy Based Blends for Additive Manufacturing by Liquid Crystal Display (LCD) Printing: The Effect of Blending and Dual Curing on Daylight Curable Resins

Claudio Tosto [1], Eugenio Pergolizzi [1], Ignazio Blanco [1], Antonella Patti [1], Paul Holt [2], Sarah Karmel [2] and Gianluca Cicala [1,*]

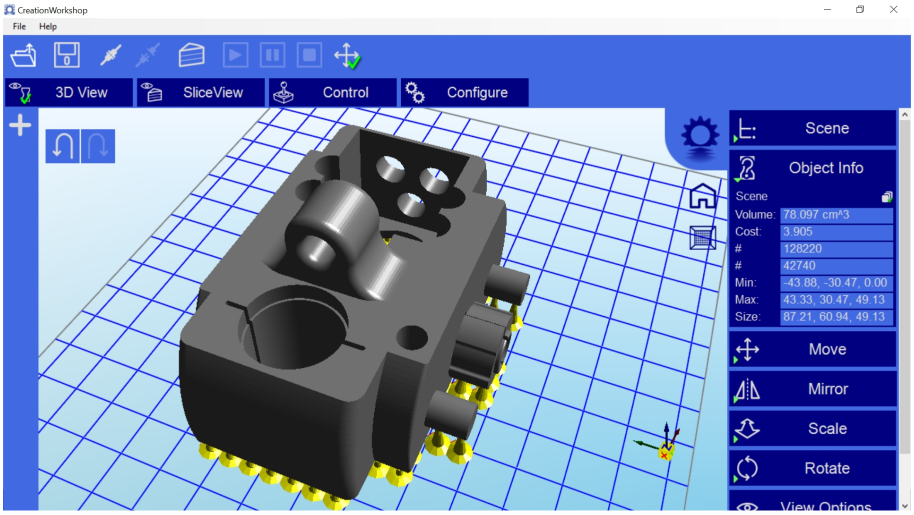

Figure S1. Sample part for LCD printing.

Table S1. Comparison of the properties of epoxy based blends with some commercial LCD/DLS-Dual Curing resin. *Producers reported HDT rather than *Tg*.

Resin Trade name	Producer	3D Technology	Tg [°C]	Viscosity [Poise]
Cyanate Ester	Carbon3D	DLS -Dual Curing	175	5
Prototyping Acrylate	Carbon3D	DLS -Dual Curing	47	-
Standard Resin	Wanhao	LCD	104	0.9
Fusion Gray	Asiga	DLP	160	-
PlasGray	Asiga	DLP	84	3.43
Basic	Zortrax	DLP	80*	1.7
PLASTCure Rigid 10 500	Prodways	DLP	125*	1.1
Cream Hard	Photocentric	LCD Daylight-Dual Cure	45	1.2
Cream Hard	Photocentric	LCD Daylight-Dual Cure (Post thermal curing - in this study)	137	1.2
CE7030	This study	LCD Daylight-Dual Cure	165	6.3
CE5050	This study	LCD Daylight-Dual Cure	174	12

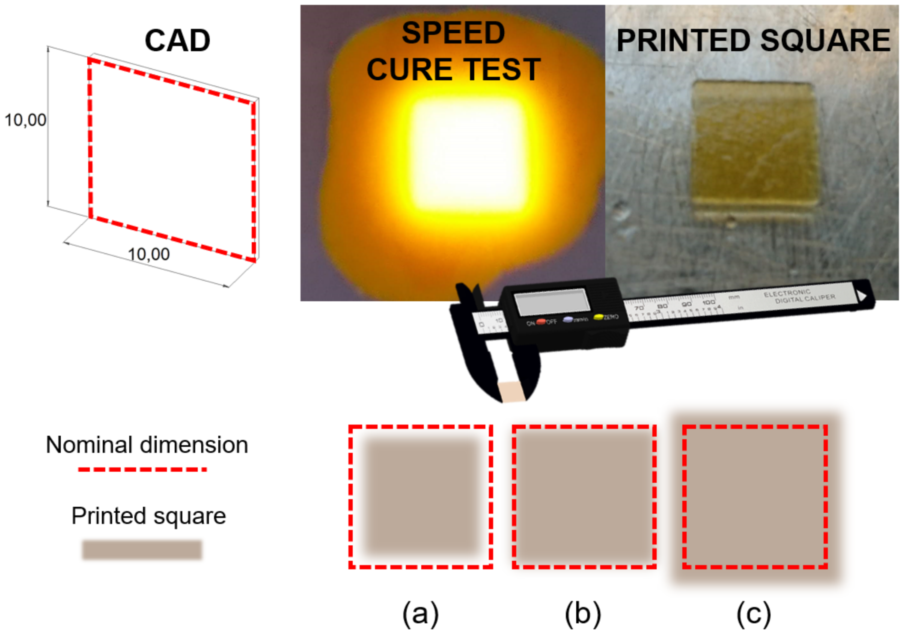

Figure S2. Width overcure: (a) negative, resin not fully cured; (b) null, properly cured resin; (c) positive, resin over cured.
